# A rare case of solitary intraosseous neurofibroma of the mandible with radiological insights

**DOI:** 10.1016/j.radcr.2024.10.130

**Published:** 2024-11-22

**Authors:** Sakshi Dudhe, Devyansh Nimodia, Gaurav V. Mishra, Pratapsingh Hanuman Parihar, Paritosh Bhangale, Anjali Kumari, Rishitha Kotla

**Affiliations:** a Department of Radiodiagnosis, Datta Meghe Institute of Medical Sciences, Sawangi, Wardha, Maharashtra, India 442001; b Department of Psychiatry, Datta Meghe Institute of Medical Sciences, Sawangi, Wardha, Maharashtra, India 442001

**Keywords:** Neurofibroma, Radiology, Tumour, Solitary, Mandible, Rare

## Abstract

Neurofibromas (NF), rare benign peripheral nerve sheath tumors, are typically linked to neurofibromatosis type 1 (NF1). This case report presents a rare instance of a neurofibroma located in the mandible of a 12-year-old male patient, who presented with localized swelling and discomfort in the lower jaw. Clinical examination revealed a firm, nontender mass on palpation. Investigations such as radiography and computed tomography were done, followed by surgical excision. This case underscores the importance of considering neurofibromas in the differential diagnosis of mandibular lesions and highlights the need for a multidisciplinary approach in management. The case adds to the literature by detailing the presentation, imaging characteristics, and treatment options for neurofibromas in the oral and maxillofacial region, underscoring the need for heightened clinical awareness of such rare occurrences.

## Introduction

NF is classified as nonmalignant neoplasm originating from peripheral nervous system. They are very rarely found within bony structures. Generally, neurofibroma manifest as an independent solitary lesion, rather than occurring in association with neurofibromatosis, an autosomal dominant genetically inherited disorder [1]. World Health Organization has defined neurofibroma as a benign tumor of the peripheral nerve sheath with a heterogenous composition of Schwann cells, perineural hybrid cells, and intraneural fibroblasts [2].

Solitary neurofibroma (NF) of oral cavity was first documented by Bruce in 1954 and very few cases have been reported after that. Neurofibromas can manifest as isolated lesions or as part of the broader condition known as neurofibromatosis, also referred to as von Recklinghausen's disease. Clinically, oral neurofibromas usually present as painless, slow-growing pediculated or sessile nodules [3].

Neurofibromas localized within the craniofacial region are known to arise from 5th, 7th,8th,9th, 11th, or 12th cranial nerves, although their occurrence in the oral cavity is rare [4]. This case report aims to present a unique instance of a neurofibroma located in the mandible of a 35-year-old female patient, detailing the clinical presentation, diagnostic workup, surgical management, and postoperative outcomes. By sharing this case, we aspire to augment clinicians' cognizance regarding the necessity of considering neurofibromas in the differential diagnosis of mandibular lesions and to emphasize the importance of a multidisciplinary approach in the management of such cases.

## Case presentation

**Fig. 1:**
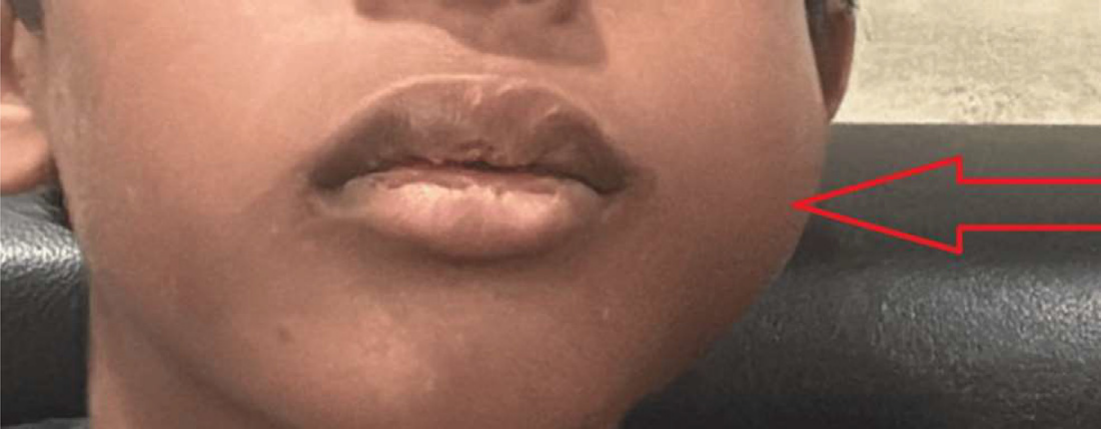
Clinical picture of the patient depicting swelling on left side of the face, particularly in the region of left mandible (red arrow).

A 12-year-old male came to surgery out-patient department with a swelling in the region of left hemimandible. The swelling was painless in nature. It was observed 2 years back by the patient's mother, which was noted as a small swelling, and hence the patient did not see any doctor for the same. Swelling was slow growing and progressed to current size of approximate size 4 × 5 cm. Externally, swelling was seen on the left side of the face, as shown in Fig. 1.

Overlying skin was normal, with no evidence of any discoloration.

**Fig. 2:**
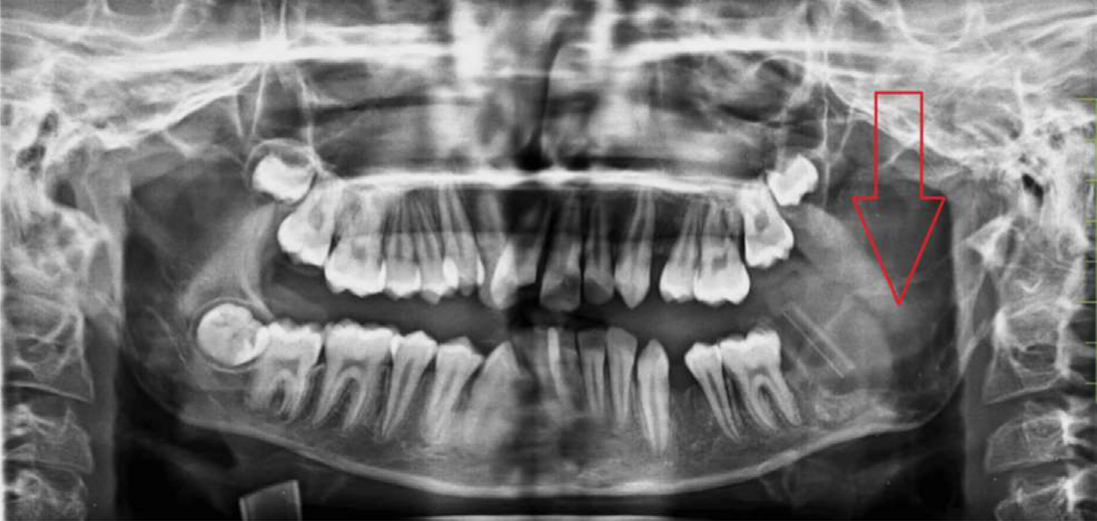
OPG image showing radiolucent lesion in left mandible (red arrow).

The swelling was rounded in shape externally, of firm consistency, with rough texture on palpation. No pain or tenderness was present on local examination. Dermatological evaluation was normal. Radiograph depicted a radiolucent area at left hemimandible as shown in Fig. 2.

**Fig. 3:**
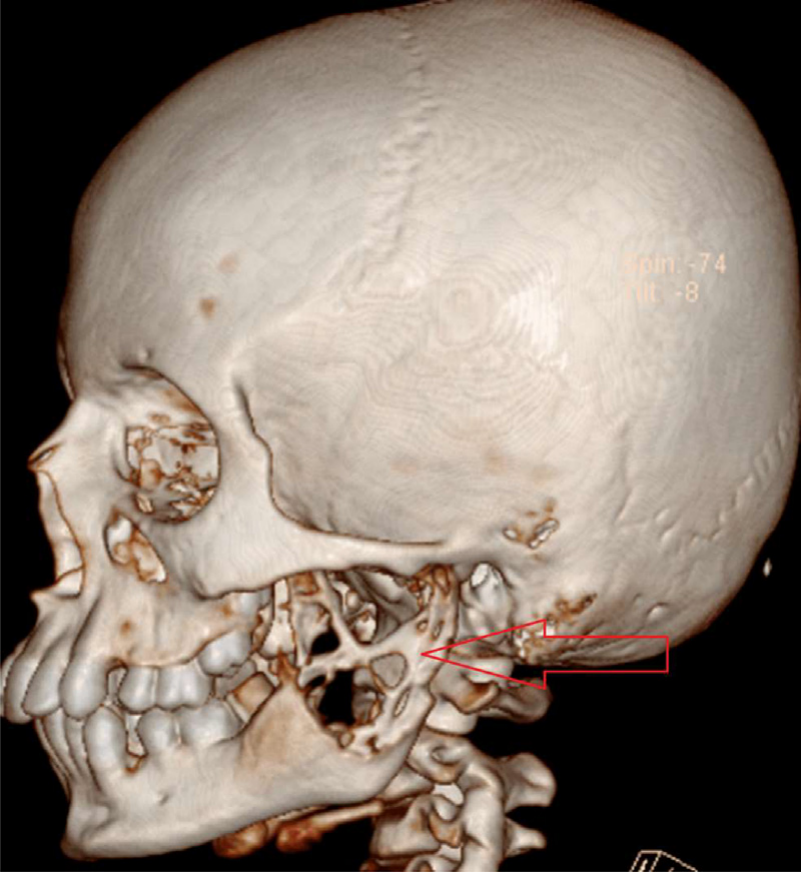
Reformatted CT scan image of the skull demonstrating expansile cystic lesion with cortical erosion in area of left hemimandible (red arrow).

**Fig. 4:**
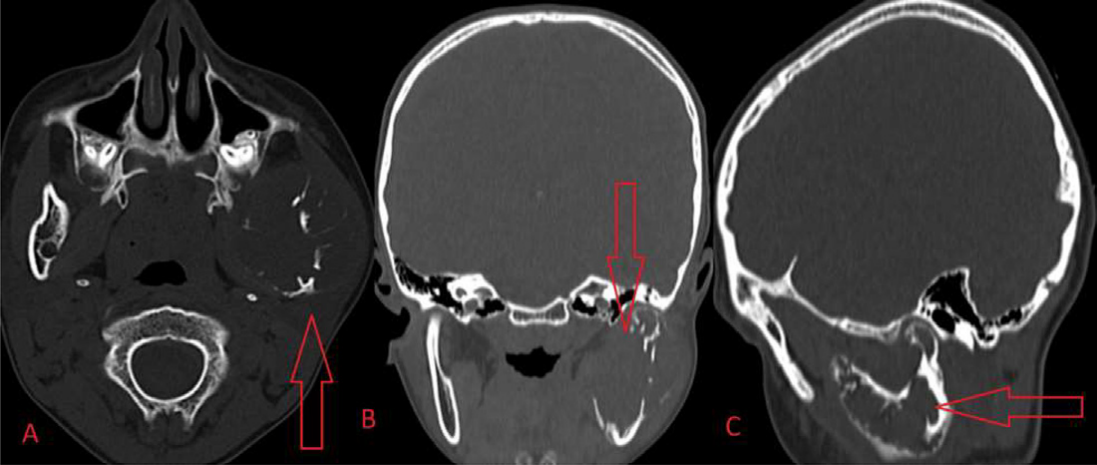
CT bone window in axial (A), coronal (B), sagittal (C) sections showing expansile osseous cystic lesion causing erosion and periosteal elevation measuring 4.6 × 4.5 × 5 cm noted involving left angle, ramus, condylar and coronoid process of left hemimandible.

**Fig. 5:**
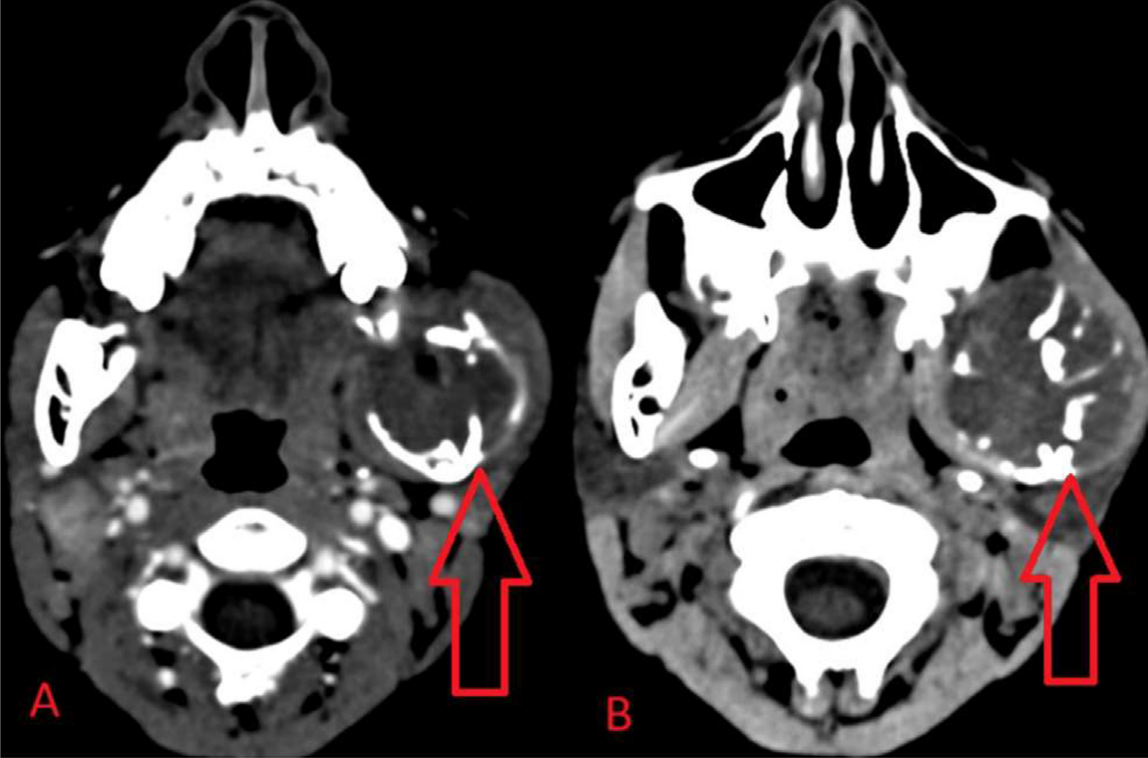
CECT soft tissue window in axial sections (A and B) showing well defined expansile lytic lesion with homogenously enhancing soft tissue component within, measuring 4.6 × 4.5 × 5 cm noted in left hemimandible (red arrows).

Ultrasound of the swelling revealed complex cystic lesion partly superficial and partly deep to ramus of left mandible, suggesting osseous cystic lesion. Computed tomography (CT) was done which depicted well defined expansile lytic lesion with homogenously enhancing soft tissue component within, noted in left angle, ramus, condylar and coronoid process of left hemimandible as shown in Fig. 3, Fig. 4, Fig. 5. The lesion measured 4.6 × 4.5 × 5 cm. Lesion had corticated boundaries with erosion of underlying bone and periosteal elevation. Drain was also put in-situ. The lesion was seen compressing the left parotid and medial pterygoid muscle, exerting mass effect on it. Radiological diagnosis of Neurofibroma of mandible was made.

The patient was advised to undergo surgery as a definitive treatment. Left hemi-mandibulectomy of the patient was done. Postoperative follow-up was done and it showed no recurrence. Patient was happy postsurgery and no local symptoms were noted.

## Discussion

Neurofibroma (NF) represents a benign nerve sheath neoplasm that can present as solitary single or multiple lesions. These lesions are seen mostly associated with neurofibromatosis type 1. Neurofibromatosis type 1 occures due to germline mutation in the NF1 gene, which is located at 17q11.2. Oral cavity neurofibromas are infrequently seen, constituting for only 6% of cases, mostly involving the tongue or buccal mucosa. Intraosseous neurofibromas of the mandible are particularly very uncommon. At initial stages these tumours are silently asymptomatic and when they grow, mass effect on adjacent vital structure sis exerted. Bone destruction is also a feature that leads to pain and lip numbness on the side of the lesion [2].

Neurofibromas most commonly involve the skin. Neurofibromas are associated with von Recklinghausen's disease and multiple endocrine neoplasia type III (MEN III). These tumours are predominantly noted in the third decade of life. Cases in age range of 10 months to 70 years of age have also been documented [3]. There is no clear evidence of sex predilection noted [5]. Neurofibroma is caused by an autosomal dominant mutation. Positive family histories have also been noted. Neurocutaneous lesions along with some soft-tissue lesions are a feature of Neurofibromatosis type 1. Bilateral acoustic neuromas are typically noted in Neurofibromatosis type 2. Neurofibromas when occurring in the head and neck region are generally solitary lesions. Instances of isolated Mandibular region neurofibromas are exceedingly [6].

Bone changes in neurofibroma of the mandible include enlargement of mandibular foramina, inferior alveolar canal, increased vertical dimension at the coronoid notch region, reduction in angle of mandible, mandibular condylar deformation, noneruption of teeth, and presence of cystic formation [7]. Inherited neurofibromas most frequently occur on the skin, with only 6% of cases reported in the oral cavity. These tumors can also develop intraosseously, with the posterior region of the mandible being the most prevalent site [1].

Neurofibroma of the mandible is more common in females. The more common location being the posterior mandible. In our case, common clinical features of neurofibromatosis like dermatological manifestations were absent. Solitary neurofibromas are benign, slow-growing tumours. These lesions are generally circumscribed and has its origin within a nerve. These lesions are composed of perineural cells, schwann cells, and mature collagen [8].

This intraosseous tumor often remains undetected for years, particularly in the absence of symptoms, and is typically discovered incidentally. In the present case, immunohistochemistry confirmed its neurogenic nature through positive for protein S-100 staining in the spindle cells that proliferate [4].The closest differential diagnosis of neurofibromas encompasses various spindle cell neoplasms, such as schwannoma, traumatic neuroma, desmoplastic melanotic melanoma, benign fibrous histiocytoma, spindle cell carcinoma, and amelanotic melanoma.

The recommended treatment for solitary neurofibroma is complete surgical excision, and recurrence is rare. However, given reports of malignant transformation in some cases, long-term patient follow-up is advised. Dentists should consider neurofibroma as a differential diagnosis when they encounter any radiolucency in the jaw region [9].

## Conclusions

This is a unique case report that gives radiological insights about the very rare entity that is linked with neurofibromatosis i.e. neurofibroma. Very few cases have been initially reported and this is one of them. If we diagnose this rare solitary intraosseous neurofibroma at an earlier stage, the guidance for clinical and surgical management becomes easy. This case makes the reader aware of considering neurofibroma of mandible, as a differential diagnosis for a radiolucent lesion found in mandible.

## Ethics approval and consent to participate

Written consent taken.

## Consent for publication

Written consent taken.

## Authors' contributions

SD and DN was involved in providing clinical details of the patient. PHP discussion on the pathology. GVM accumulated the results of the patient's radiological investigations. PB, AK and RK was involved in collecting images and formatting data. All authors have read and approved the manuscript.

## Patient consent

Informed and written consent was obtained from the patient.
